# (*S*)-1-Methoxy­carbonyl-3-(4-nitro­phen­yl)propan-2-aminium bromide

**DOI:** 10.1107/S1600536809034904

**Published:** 2009-09-09

**Authors:** Bo Wang

**Affiliations:** a Ordered Matter Science Research Center, College of Chemistry and Chemical Engineering, Southeast University, Nanjing 210096, People’s Republic of China

## Abstract

In the crystal structure of the title compound, C_10_H_13_N_2_O_4_^+^·Br^−^, inter­molecular N—H⋯Br and N—H⋯(O,Br) hydrogen bonds link the cations and anions into a two-dimensional network parallel to the *ab* plane.

## Related literature

For applications of metal-organic coordination compounds, see: Xiong *et al.* (1999 ▶); Fu, Zhang *et al.* (2008 ▶); Fu & Xiong (2008 ▶). For metal-organic frameworks with amino acid deriv­atives, see: Chen *et al.* (2000 ▶); Xie *et al.* (2002 ▶); Fu *et al.* (2007 ▶). 
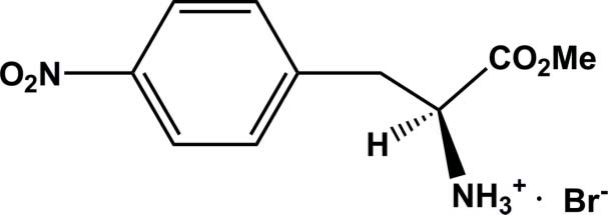


## Experimental

### 

#### Crystal data

C_10_H_13_N_2_O_4_^+^·Br^−^
*M* *_r_* = 305.13
Monoclinic, 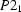

*a* = 4.9323 (10) Å
*b* = 8.6233 (17) Å
*c* = 15.226 (3) Å
β = 95.77 (3)°
*V* = 644.3 (2) Å^3^
*Z* = 2
Mo *K*α radiation
μ = 3.20 mm^−1^
*T* = 298 K
0.40 × 0.05 × 0.05 mm

#### Data collection

Rigaku Mercury2 diffractometer
Absorption correction: multi-scan (*CrystalClear*; Rigaku, 2005 ▶) *T*_min_ = 0.90, *T*_max_ = 1.00
6658 measured reflections
2917 independent reflections
2532 reflections with *I* > 2σ(*I*)
*R*_int_ = 0.055

#### Refinement

*R*[*F* ^2^ > 2σ(*F* ^2^)] = 0.042
*wR*(*F* ^2^) = 0.085
*S* = 1.04
2917 reflections
154 parameters
1 restraint
H-atom parameters constrained
Δρ_max_ = 0.62 e Å^−3^
Δρ_min_ = −0.30 e Å^−3^
Absolute structure: Flack (1983 ▶), 1202 Friedel pairs
Flack parameter: 0.008 (5)

### 

Data collection: *CrystalClear* (Rigaku, 2005 ▶); cell refinement: *CrystalClear*; data reduction: *CrystalClear*; program(s) used to solve structure: *SHELXS97* (Sheldrick, 2008 ▶); program(s) used to refine structure: *SHELXL97* (Sheldrick, 2008 ▶); molecular graphics: *SHELXTL/PC* (Sheldrick, 2008 ▶); software used to prepare material for publication: *SHELXTL/PC*.

## Supplementary Material

Crystal structure: contains datablocks I, global. DOI: 10.1107/S1600536809034904/cv2604sup1.cif

Structure factors: contains datablocks I. DOI: 10.1107/S1600536809034904/cv2604Isup2.hkl

Additional supplementary materials:  crystallographic information; 3D view; checkCIF report

## Figures and Tables

**Table 1:** Hydrogen-bond geometry (Å, °)

*D*—H⋯*A*	*D*—H	H⋯*A*	*D*⋯*A*	*D*—H⋯*A*
N1—H1*A*⋯O2^i^	0.87	2.61	3.031 (4)	111
N1—H1*A*⋯Br1^ii^	0.87	2.61	3.290 (3)	135
N1—H1*B*⋯Br1^iii^	0.93	2.51	3.303 (3)	143
N1—H1*C*⋯Br1^iv^	1.00	2.55	3.495 (3)	157

Symmetry codes: (i) 
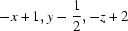
; (ii) 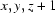; (iii) 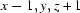; (iv) 
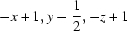
.

**Fig. 1.**
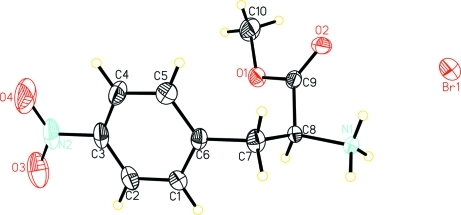
A view of the title compound with the atomic numbering scheme. Displacement ellipsoids are drawn at the 30% probability level.

**Fig. 2.**
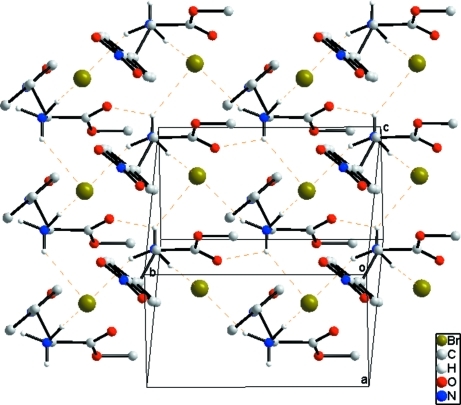
A portion of the crystal packing, viewed along the c axis. Dashed lines denote N—H···Br and N—H···O hydrogen bonds. H atoms not involved in hydrogen bonding have been omitted for clarity.

## supplementary crystallographic information

### Comment

The construction of metal-organic coordination compounds has attracted much attention owing to potential functions, such as permittivity, fluorescence, magnetism and optical properties (Fu, Zhang *et al.*, 2008; Xiong *et al.*, 1999; Fu & Xiong, 2008). Amino acid derivatives constitute a class of excellent ligands for the construction of novel metal-organic frameworks (Fu *et al.*, 2007; Xie *et al.*, 2002; Chen *et al.*, 2000). We report here the crystal structure of the title compound.

The title compound is built up from a Br^-^ anion and a protonated amino group cation (Fig.1). The nitro group and the benzene ring are nearly coplanar being twisted to each other by 2.39 (6)°. The *S* absolute configuration at C8 is deduced from the synthetic pathway and confirmed by the X-ray analysis.

The crystal packing is stabilized by N—H···Br and N—H···O H-bonds (Table 1) building an infinite two-dimensional network parallel to *ab* plane (Fig.2).

### Experimental

Under nitrogen protection, methyl 2-amino-3-(4-nitrophenyl)propanoate (30 mmol), nitric acid (50 mmol) and sulfuric acid (20 mmol) were added in a flask. The mixture was stirred at 110 °C for 3 hours. The resulting solution was poured into ice water (100mL), then filtered and washed with distilled water. The crude product was recrystallized with distilled water by adding 4ml HBr to yield colourless needle-like crystals, suitable for X-ray analysis.

### Refinement

C-bound H atoms were positioned geometrically and treated as riding, with C-H = 0.93 Å (aromatic), C-H = 0.96 Å (methyl), C-H = 0.97 Å (methylene) and C-H = 0.98 Å (methine), with *U*_iso_(H) = 1.2*U*_eq_(C) and *U*_iso_(H) = 1.5*U*_eq_(methyl). The H atoms of amine group were located in difference Fourier maps and at the last stage of refinement they were treated as riding, with *U*_iso_(H) = 1.5*U*_eq_(N).

### Crystal data

**Table:**

C_10_H_13_N_2_O_4_^+^·Br^−^	*F*(000) = 308
*M**_r_* = 305.13	*D*_x_ = 1.573 Mg m^−^^3^
Monoclinic, *P*2_1_	Mo *K*α radiation, λ = 0.71073 Å
Hall symbol: P 2yb	Cell parameters from 2532 reflections
*a* = 4.9323 (10) Å	θ = 3.6–27.5°
*b* = 8.6233 (17) Å	µ = 3.20 mm^−^^1^
*c* = 15.226 (3) Å	*T* = 298 K
β = 95.77 (3)°	Needle, colourless
*V* = 644.3 (2) Å^3^	0.40 × 0.05 × 0.05 mm
*Z* = 2	

### Data collection

**Table:**

Rigaku Mercury2 diffractometer	2917 independent reflections
Radiation source: fine-focus sealed tube	2532 reflections with *I* > 2σ(*I*)
graphite	*R*_int_ = 0.055
Detector resolution: 13.6612 pixels mm^-1^	θ_max_ = 27.5°, θ_min_ = 3.6°
CCD profile fitting scans	*h* = −6→6
Absorption correction: multi-scan (*CrystalClear*; Rigaku, 2005)	*k* = −11→11
*T*_min_ = 0.90, *T*_max_ = 1.00	*l* = −19→19
6658 measured reflections	

### Refinement

**Table:**

Refinement on *F*^2^	Secondary atom site location: difference Fourier map
Least-squares matrix: full	Hydrogen site location: inferred from neighbouring sites
*R*[*F*^2^ > 2σ(*F*^2^)] = 0.042	H-atom parameters constrained
*wR*(*F*^2^) = 0.085	*w* = 1/[σ^2^(*F*_o_^2^)]
*S* = 1.03	(Δ/σ)_max_ < 0.001
2917 reflections	Δρ_max_ = 0.62 e Å^−^^3^
154 parameters	Δρ_min_ = −0.30 e Å^−^^3^
1 restraint	Absolute structure: Flack (1983), 1202 Friedel pairs
Primary atom site location: structure-invariant direct methods	Flack parameter: 0.008 (5)

### Special details

**Table:**

Geometry. All esds (except the esd in the dihedral angle between two l.s. planes) are estimated using the full covariance matrix. The cell esds are taken into account individually in the estimation of esds in distances, angles and torsion angles; correlations between esds in cell parameters are only used when they are defined by crystal symmetry. An approximate (isotropic) treatment of cell esds is used for estimating esds involving l.s. planes.
Refinement. Refinement of F^2^ against ALL reflections. The weighted R-factor wR and goodness of fit S are based on F^2^, conventional R-factors R are based on F, with F set to zero for negative F^2^. The threshold expression of F^2^ > 2sigma(F^2^) is used only for calculating R-factors(gt) etc. and is not relevant to the choice of reflections for refinement. R-factors based on F^2^ are statistically about twice as large as those based on F, and R- factors based on ALL data will be even larger.

### Fractional atomic coordinates and isotropic or equivalent isotropic displacement parameters (Å^2^)

**Table:**

	*x*	*y*	*z*	*U*_iso_*/*U*_eq_	
C6	0.4984 (8)	0.4001 (4)	0.7127 (2)	0.0390 (9)	
C2	0.8039 (10)	0.2806 (6)	0.6203 (3)	0.0565 (12)	
H2	0.9359	0.2063	0.6122	0.068*	
C7	0.3794 (8)	0.4113 (5)	0.7995 (2)	0.0438 (10)	
H7A	0.3349	0.3081	0.8188	0.053*	
H7B	0.2118	0.4708	0.7916	0.053*	
C3	0.7186 (9)	0.3798 (6)	0.5544 (3)	0.0527 (12)	
C5	0.4128 (9)	0.5000 (5)	0.6421 (3)	0.0554 (11)	
H5	0.2796	0.5742	0.6489	0.067*	
C4	0.5236 (10)	0.4886 (6)	0.5644 (3)	0.0642 (15)	
H4	0.4667	0.5550	0.5180	0.077*	
C1	0.6940 (9)	0.2910 (5)	0.6986 (3)	0.0481 (11)	
H1	0.7523	0.2226	0.7439	0.058*	
O1	0.7970 (5)	0.6832 (4)	0.79876 (16)	0.0488 (7)	
C9	0.6074 (6)	0.6629 (6)	0.85319 (19)	0.0360 (7)	
O2	0.4710 (6)	0.7600 (3)	0.88109 (18)	0.0495 (7)	
C8	0.5764 (8)	0.4886 (4)	0.8712 (2)	0.0325 (8)	
H8	0.7551	0.4384	0.8731	0.039*	
N2	0.8359 (11)	0.3691 (6)	0.4697 (3)	0.0759 (13)	
O3	1.0072 (9)	0.2696 (7)	0.4603 (2)	0.1077 (17)	
O4	0.7514 (11)	0.4561 (6)	0.4108 (3)	0.1200 (18)	
C10	0.8330 (12)	0.8435 (6)	0.7701 (3)	0.0717 (15)	
H10A	0.9736	0.8472	0.7308	0.107*	
H10B	0.8837	0.9076	0.8206	0.107*	
H10C	0.6653	0.8808	0.7399	0.107*	
N1	0.4679 (6)	0.4728 (4)	0.95973 (18)	0.0391 (8)	
H1A	0.6015	0.4736	1.0021	0.059*	
H1B	0.3245	0.5428	0.9591	0.059*	
H1C	0.3835	0.3672	0.9607	0.059*	
Br1	0.99538 (6)	0.66497 (4)	0.05395 (2)	0.04632 (13)	

### Atomic displacement parameters (Å^2^)

**Table:**

	*U*^11^	*U*^22^	*U*^33^	*U*^12^	*U*^13^	*U*^23^
C6	0.045 (2)	0.043 (2)	0.029 (2)	−0.0124 (19)	0.0045 (17)	−0.0031 (18)
C2	0.065 (3)	0.061 (3)	0.045 (3)	0.003 (2)	0.009 (2)	−0.018 (2)
C7	0.042 (2)	0.050 (2)	0.041 (2)	−0.014 (2)	0.0121 (18)	−0.006 (2)
C3	0.064 (3)	0.064 (3)	0.032 (2)	−0.019 (2)	0.015 (2)	−0.014 (2)
C5	0.062 (3)	0.056 (3)	0.049 (3)	0.007 (2)	0.006 (2)	0.002 (2)
C4	0.096 (4)	0.064 (3)	0.033 (3)	−0.005 (3)	0.010 (3)	0.008 (2)
C1	0.063 (3)	0.041 (2)	0.040 (2)	0.003 (2)	0.005 (2)	−0.0061 (19)
O1	0.0659 (16)	0.0393 (17)	0.0455 (14)	−0.0094 (17)	0.0266 (12)	−0.0001 (15)
C9	0.0413 (16)	0.0386 (16)	0.0280 (16)	−0.001 (3)	0.0032 (13)	0.001 (2)
O2	0.0603 (18)	0.0379 (16)	0.0533 (18)	0.0130 (15)	0.0201 (14)	0.0078 (14)
C8	0.039 (2)	0.0319 (19)	0.0278 (19)	0.0033 (17)	0.0099 (15)	−0.0005 (16)
N2	0.098 (4)	0.094 (3)	0.039 (3)	−0.028 (3)	0.024 (3)	−0.021 (2)
O3	0.109 (4)	0.151 (5)	0.070 (3)	−0.003 (3)	0.044 (3)	−0.034 (3)
O4	0.177 (5)	0.142 (4)	0.049 (3)	−0.017 (4)	0.048 (3)	0.001 (3)
C10	0.103 (4)	0.058 (3)	0.059 (3)	−0.015 (3)	0.031 (3)	0.010 (2)
N1	0.054 (2)	0.0350 (17)	0.0299 (17)	0.0033 (15)	0.0137 (15)	0.0039 (14)
Br1	0.0471 (2)	0.0436 (2)	0.0490 (2)	0.0020 (3)	0.00881 (15)	−0.0088 (3)

### Geometric parameters (Å, °)

**Table:**

C6—C1	1.380 (6)	O1—C9	1.322 (4)
C6—C5	1.408 (5)	O1—C10	1.466 (6)
C6—C7	1.503 (5)	C9—O2	1.180 (5)
C2—C3	1.353 (6)	C9—C8	1.538 (6)
C2—C1	1.363 (5)	C8—N1	1.506 (4)
C2—H2	0.9300	C8—H8	0.9800
C7—C8	1.538 (5)	N2—O4	1.210 (6)
C7—H7A	0.9700	N2—O3	1.223 (6)
C7—H7B	0.9700	C10—H10A	0.9600
C3—C4	1.363 (6)	C10—H10B	0.9600
C3—N2	1.468 (6)	C10—H10C	0.9600
C5—C4	1.356 (5)	N1—H1A	0.8742
C5—H5	0.9300	N1—H1B	0.9289
C4—H4	0.9300	N1—H1C	1.0023
C1—H1	0.9300		
			
C1—C6—C5	117.4 (4)	O2—C9—O1	126.6 (5)
C1—C6—C7	121.4 (4)	O2—C9—C8	124.0 (3)
C5—C6—C7	121.2 (4)	O1—C9—C8	109.3 (4)
C3—C2—C1	119.1 (4)	N1—C8—C9	107.4 (3)
C3—C2—H2	120.4	N1—C8—C7	109.9 (3)
C1—C2—H2	120.4	C9—C8—C7	111.4 (3)
C6—C7—C8	112.1 (3)	N1—C8—H8	109.4
C6—C7—H7A	109.2	C9—C8—H8	109.4
C8—C7—H7A	109.2	C7—C8—H8	109.4
C6—C7—H7B	109.2	O4—N2—O3	122.6 (5)
C8—C7—H7B	109.2	O4—N2—C3	118.3 (6)
H7A—C7—H7B	107.9	O3—N2—C3	119.0 (5)
C2—C3—C4	121.5 (4)	O1—C10—H10A	109.5
C2—C3—N2	119.4 (5)	O1—C10—H10B	109.5
C4—C3—N2	119.1 (5)	H10A—C10—H10B	109.5
C4—C5—C6	120.3 (4)	O1—C10—H10C	109.5
C4—C5—H5	119.8	H10A—C10—H10C	109.5
C6—C5—H5	119.8	H10B—C10—H10C	109.5
C5—C4—C3	119.9 (4)	C8—N1—H1A	110.5
C5—C4—H4	120.1	C8—N1—H1B	105.7
C3—C4—H4	120.1	H1A—N1—H1B	121.3
C2—C1—C6	121.8 (4)	C8—N1—H1C	106.3
C2—C1—H1	119.1	H1A—N1—H1C	106.2
C6—C1—H1	119.1	H1B—N1—H1C	105.9
C9—O1—C10	115.2 (4)		
			
C1—C6—C7—C8	75.9 (5)	C10—O1—C9—O2	−1.2 (5)
C5—C6—C7—C8	−104.6 (4)	C10—O1—C9—C8	175.3 (3)
C1—C2—C3—C4	−0.6 (7)	O2—C9—C8—N1	−29.0 (4)
C1—C2—C3—N2	−179.9 (4)	O1—C9—C8—N1	154.3 (3)
C1—C6—C5—C4	−0.8 (6)	O2—C9—C8—C7	91.3 (4)
C7—C6—C5—C4	179.7 (4)	O1—C9—C8—C7	−85.4 (3)
C6—C5—C4—C3	0.1 (7)	C6—C7—C8—N1	−170.5 (3)
C2—C3—C4—C5	0.6 (7)	C6—C7—C8—C9	70.7 (4)
N2—C3—C4—C5	179.9 (4)	C2—C3—N2—O4	178.0 (5)
C3—C2—C1—C6	−0.2 (7)	C4—C3—N2—O4	−1.3 (7)
C5—C6—C1—C2	0.9 (6)	C2—C3—N2—O3	0.6 (7)
C7—C6—C1—C2	−179.7 (4)	C4—C3—N2—O3	−178.7 (5)

### Hydrogen-bond geometry (Å, °)

**Table:**

*D*—H···*A*	*D*—H	H···*A*	*D*···*A*	*D*—H···*A*
N1—H1A···O2^i^	0.87	2.61	3.031 (4)	111
N1—H1A···Br1^ii^	0.87	2.61	3.290 (3)	135
N1—H1B···Br1^iii^	0.93	2.51	3.303 (3)	143
N1—H1C···Br1^iv^	1.00	2.55	3.495 (3)	157

Symmetry codes: (i) −*x*+1, *y*−1/2, −*z*+2; (ii) *x*, *y*, *z*+1; (iii) *x*−1, *y*, *z*+1; (iv) −*x*+1, *y*−1/2, −*z*+1.
